# The Double-Edged Sword in Pathogenic Trypanosomatids: The Pivotal Role of Mitochondria in Oxidative Stress and Bioenergetics

**DOI:** 10.1155/2014/614014

**Published:** 2014-03-31

**Authors:** Rubem Figueiredo Sadok Menna-Barreto, Solange Lisboa de Castro

**Affiliations:** Laboratório de Biologia Celular, Instituto Oswaldo Cruz, Fundação Oswaldo Cruz, Avenida Brasil 4365, 21040-360 Manguinhos, RJ, Brazil

## Abstract

The pathogenic trypanosomatids *Trypanosoma brucei*, *Trypanosoma cruzi*, and *Leishmania* spp. are the causative agents of African trypanosomiasis, Chagas disease, and leishmaniasis, respectively. These diseases are considered to be neglected tropical illnesses that persist under conditions of poverty and are concentrated in impoverished populations in the developing world. Novel efficient and nontoxic drugs are urgently needed as substitutes for the currently limited chemotherapy. Trypanosomatids display a single mitochondrion with several peculiar features, such as the presence of different energetic and antioxidant enzymes and a specific arrangement of mitochondrial DNA (kinetoplast DNA). Due to mitochondrial differences between mammals and trypanosomatids, this organelle is an excellent candidate for drug intervention. Additionally, during trypanosomatids' life cycle, the shape and functional plasticity of their single mitochondrion undergo profound alterations, reflecting adaptation to different environments. In an uncoupling situation, the organelle produces high amounts of reactive oxygen species. However, these species role in parasite biology is still controversial, involving parasite death, cell signalling, or even proliferation. Novel perspectives on trypanosomatid-targeting chemotherapy could be developed based on better comprehension of mitochondrial oxidative regulation processes.

## 1. Trypanosomatids and Diseases

Among trypanosomatids, there are several pathogenic species:* Trypanosoma brucei*, the causative agent of African trypanosomiasis;* Trypanosoma cruzi*, of Chagas disease; and* Leishmania* spp., of leishmaniasis. These diseases, with high morbidity and mortality rates, affect millions of impoverished populations in the developing world, display a limited response to chemotherapy, and are classified as neglected tropical diseases by the World Health Organization [1].

Trypanosomatids exhibit the most typical eukaryotic organelles such as plasma membrane, endoplasmic reticulum, and Golgi; however, some particular structures are also presented. Immediately below the plasma membrane, there is a structural cage of stable microtubules called subpellicular microtubules. The flagellum originated from a flagellar pocket presenting a typical axoneme and a paraflagellar rod, structures involved in the flagellum beating. The nucleus is single maintaining the integrity of its envelope during the whole mitosis [2]. Glycosomes are peroxisomes-like organelles exclusive of trypanosomatids, where it was also compartmentalized part of glycolytic pathway as well as lipids and amino acids oxidation enzymes [3]. Another peculiar structure is acidocalcisome, acidic electron-dense organelle involved in polyphosphate and pyrophosphate metabolism that also works as ions storage [4]. As it will be described in item Section 3.1, the mitochondrial morphology in trypanosomatids is unique presenting a characteristic architecture. These protozoa belong to the earliest diverging branches of the eukaryotic evolutionary tree which have mitochondria, fact that reflects in the mitochondrial organization. The topology of DNA network together with the functionality of the maxicircles and minicircles led to peculiar events in this organelle biogenesis, despite the similarities between mitochondrial genome of trypanosomatids and other eukaryotes. Some mitochondrial genes named cryptogenes presented unusual structure, being the transcripts remodeling by an RNA editing process [5].

Human African trypanosomiasis (HAT) or sleeping sickness is caused by* T. brucei *and can be fatal if not treated. In 2009, after continued control efforts, the number of reported cases dropped below 10,000 for the first time in 50 years; presently, the estimated number of cases is currently 30,000, and 70 million people are at risk of HAT [6]. This disease is transmitted by the bite of certain species of the genus* Glossina* (tsetse flies), found only in sub-Saharan Africa. HAT occurs in two clinical forms: chronic caused by* T. brucei gambiense* (mostly in West and Central Africa) that accounts for more than 98% of reported cases and acute caused by* T. brucei rhodesiense *(mainly in East and South Central Africa). The acute disease (stage 1) is characterized by the presence of the parasites in the vasculature and lymphatic systems. Without treatment, the parasites penetrate the blood-brain barrier and invade the central nervous system initiating chronic stage (stage 2) that manifests as mental disturbances, anxiety, hallucinations, slurred speech, seizures, and difficulty in walking and talking [7]. These problems can develop over many years in the gambiense form and over several months in the rhodesiense form. The type of chemotherapeutic treatment depends on the stage of the disease, that is, on the degree of central nervous system involvement and the consequent pharmacological need to breach the blood-brain barrier reaching the parasite [8]. The drugs used in the first stage are of lower toxicity and are easier to administer, with pentamidine for infections by* T. b. gambiense* and suramin for* T. b. rhodesiense*. In this case,* T. b. rhodesiense* infections are treated with melarsoprol, while* T. b. gambiense* infections are treated with either eflornithine or a nifurtimox/eflornithine combination therapy (NECT). However, none of these treatments are ideal. Melarsoprol is extremely toxic and has increasing treatment failures. Eflornithine is expensive, is laborious to administer, and lacks efficacy against* T. b. rhodesiense*. The development of NECT reduced the i.v. infusions of eflornithine but it is not ideal, since parenteral administration is still required and patients must be hospitalized for the duration of treatment.

Chagas disease is caused by the protozoan* T. cruzi* and affects approximately eight million individuals in Latin America, of whom 30–40% either have or will develop cardiomyopathy, digestive megasyndromes, or both [9]. Although vectorial (*Triatoma infestans*) and transfusional transmissions have steadily declined [10], this disease can also be orally transmitted through the ingestion of contaminated food or liquids. More recently, a major concern has been the emergence of Chagas disease in nonendemic areas, such as North America and Europe, due to the immigration of infected individuals [11]. Chagas disease is characterised by two clinical phases: a short, acute phase defined by patent parasitaemia and a long, progressive chronic phase [12]. The available chemotherapy for this illness includes two nitroheterocyclic agents, nifurtimox and benznidazole, which are effective against acute infections but show poor activity in the late chronic phase, with severe collateral effects and limited efficacy against different parasitic isolates. These drawbacks justify the urgent need to identify better drugs to treat chagasic patients [13].

Leishmaniasis, which is caused by different species of* Leishmania* with an estimated 12 million cases worldwide, being the infection caused by the bite of infected female sandflies of the genera* Phlebotomus* (Europe, Asia, Africa) and* Lutzomyia* (America) [14]. In VL,* Leishmania donovani* and* Leishmania infantum* (equivalent to* Leishmania chagasi* in South America), being different pathologies associated with these species.* L. donovani* causes distinct pathologies in India and Sudan as well as some strains of* L. infantum* can cause CL. The post-treatment some* L. donovani*-infected patients develop into the diffuse cutaneous form (DCL) named post-kala-azar dermal leishmaniasis (PKDL) [15]. CL also presents in patients in many different forms, though most patients have limited self-cured cutaneous lesions. Over 15 species of* Leishmania* cause CL in humans, with species such as* Leishmania major*,* Leishmania tropica,* and* Leishmania aethiopica* in the Old World and* Leishmania mexicana*,* Leishmania amazonensis*,* Leishmania braziliensis*,* Leishmania panamensis,* and* Leishmania guyanensis* in the New World. Pentostam and Glucantime are first-line drugs for both VL and CL; however, they present several limitations, including severe side effects, the need for daily parenteral administration, and the development of drug resistance. Amphotericin B, normally considered a second-line drug, has been the first line in Bihar (India) for VL following the loss of effectiveness of antimonial drugs. The Amphotericin B formulation AmBisome, the aminoglycoside paromomycin, and the phospholipid analogue miltefosine (oral administration) have been registered for the treatment of VL. On the other hand, for CL, besides antimonials, there are limited proven treatments, that is, pentamidine, amphotericin B, and miltefosine to specific types in South America and paromomycin, only as topical formulation [16, 17].

## 2. Mitochondria in Higher Eukaryotes

The mitochondrion is a membrane-bound organelle responsible for energy production is involved in growth, differentiation, calcium homeostasis, redox balance, the stress response, and death [18, 19]. The compartmentalised organisation of the mitochondrion in inner and outer membranes, intermembrane space, and the matrix provides an optimal microenvironment for many other biosynthetic and catabolic pathways, such as *β*-oxidation, heme biosynthesis, steroidogenesis, gluconeogenesis, and amino acid metabolism [20].

Mitochondrial shape and positioning in cells are tightly regulated by fission and fusion events, and an imbalance between these events can lead to shifts in the morphology and viability of the organelle [21]. Fission is required for organelle biogenesis and for the removal of aged or damaged mitochondria through autophagy (mitophagy), allowing organelle content to be degraded or recycled. Fusion is a two-step process in which the outer and inner membranes fuse by separate events. In mammals, outer membrane fusion is controlled by the GTPase mitofusin (Mfn 1 and 2), whereas inner membrane fusion is controlled by optic atrophy OPA 1, a dynamin-like protein responsible for the maintenance of crista morphology [21].

The mitochondrial precursor proteins are synthesised in the cytosol by free ribosomes and must be imported into the organelle by translocases present in the outer and inner mitochondrial membranes [22]. Signal peptides and specific chaperones direct these precursors to the target compartment. The translocase of the outer membrane (TOM) complex is responsible for the first recognition, and the translocase of the inner membrane (TIM) complex is involved in the import of the cleavable preproteins into the organelle matrix. Additionally, OXA complex helps TOM in the insertion of inner membrane proteins and sorting and assembly machinery (SAM) complex is involved in the assembly of *β*-barrel proteins into the outer mitochondrial membrane [23].

In response to changes in the intracellular environment by different stress signals, such as a loss of growth factors, hypoxia, oxidative stress, and DNA damage, mitochondria become producers of excessive reactive oxygen species (ROS) and release prodeath proteins, resulting in disrupted ATP synthesis and the activation of cell death pathways [24]. The switch to apoptotic cell death is mediated by cysteine proteases named caspases, which cleave strategic substrates. Another important step in the apoptotic pathway is the permeabilisation of the outer mitochondrial membrane, leading the release of proapoptotic proteins. During stress, both autophagy and apoptosis are activated, and enhanced mitophagy is an early response that promotes survival by removing damaged mitochondria. With increased mitochondrial injury, apoptosis becomes dominant, and inactivation of critical proteins of the autophagic pathway leads to cell death [25].

## 3. Mitochondria in Trypanosomatids

### 3.1. Ultrastructural Architecture and Mitochondrial Dynamics

The most remarkable morphological difference between the mitochondria of higher eukaryotes and trypanosomatids is the number and relative volume of the organelles. Thousands of mitochondria can be detected in mammalian cells, representing nearly 20% of the total cellular volume, whereas only a single and ramified organelle is observed in the parasites [26]. This peculiar ultrastructural characteristic was confirmed in all* T. cruzi* developmental forms by 3D reconstruction [27, 28], and the hypothesis was extended to other pathogenic trypanosomatids.

**Figure 1:**
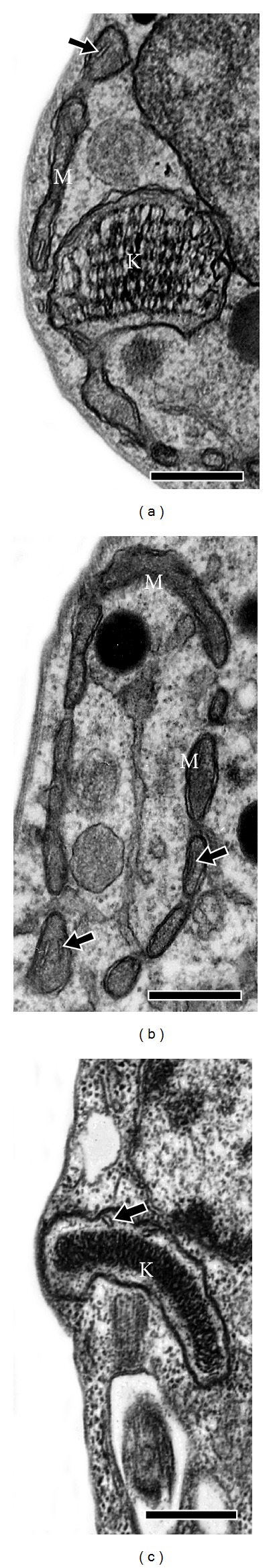
Ultrastructural analysis of the trypanosomatid mitochondrion.* T. cruzi *bloodstream trypomastigote (a) and epimastigotes (b and c). The organelle presents an elongated aspect (M), showing rare cristae (arrows). Differences in kinetoplast morphology (K) can also be observed. Bars: 400 nm.

The mitochondrial distribution varies according to the parasite and its developmental form. Generally, the organelle is elongated close to the subpellicular microtubules and the plasma membrane surrounding the entire cell and is dilated only in a disk-shaped structure called the kinetoplast (Figure 1). The ultrastructural aspect of the kinetoplast network in* T. cruzi* trypomastigotes is rounded, differing from all other species and developmental stages that present a bar shape in ultrathin sections. The morphology of the cristae and matrix is also variable, being irregularly distributed in most of the species [29, 30]. The relative volume of the entire organelle directly depends on nutrient availability, reaching 12% of the protozoan volume [30]. As occurred in other eukaryotes, the mitochondrion of trypanosomatids is very dynamic, changing its shape and function in response to the host environment, and changes in bioenergetics metabolism affect the organelle morphology [21]. As described above for other eukaryotic cells, this mitochondrial remodelling is orchestrated by fission and fusion processes and/or autophagy [31]. Despite the morphological evidence reported, the molecular mechanisms involved in the mitophagic process in protozoa are unknown [32]. However, the presence of a dynamin-like protein (DLP) has been detected in* T. brucei* and* L. major* and is related to the fission step, as in mammals [21], and to subsequent organelle segregation during mitosis [33]. To ensure correct segregation, the kDNA network is physically connected to basal bodies through a cluster of filaments that cross the kinetoplast outer and inner membranes [5]. Furthermore, BLAST analysis indicates that DLP is highly conserved in pathogenic trypanosomatids (data not shown). To date, Mtn, the main mitochondrial fusion component, has not been detected in this protozoan family, reinforcing the 3D models of a single organelle proposed by Paulin [27].

In all eukaryotes, including trypanosomatids, a large proportion of mitochondrial proteins are encoded in nuclear genes. However, after transcription, these molecules have to be translocated by the TOM, TIM, SAM, and OXA complexes from the cytosol to the organelle [34], although such complexes are poorly characterised in protists. In trypanosomatids, these translocases were first assessed in* T. brucei*, in which the essential complex TOM40 is replaced by an archaic translocase named pATOM36, responsible for at least part of the import of mitochondrial matrix proteins [35]. Moreover, tbTIM50 and tbTIM17 were described recently [36], but the exact molecular mechanisms involved in mitochondrial protein import in trypanosomatids are still unknown. Interestingly, several pieces of evidence, including data on characterisation of the mitochondria protein-import machinery, suggest that trypanosomatids are among the earliest diverging eukaryotes to have mitochondria [37].

### 3.2. Molecular Structure and Function of the kDNA Network

The most peculiar characteristic of trypanosomatids is DNA organisation in the kinetoplast. In these protozoa, the mitochondrial genome consists of a complex network of interlocked DNA rings subdivided into two classes: maxicircles and minicircles, representing approximately 30% of the total cellular genome [5, 30]. The kDNA composition varies depending on the species. Approximately, several thousand minicircles and a few dozen maxicircles can be observed per organelle, with only 10% of the entire network mass composed of maxicircles [5, 38].

Maxicircles correspond to mitochondrial DNA in other eukaryotes and encode several genes of respiratory chain complexes, such as cytochrome oxidase, NADH dehydrogenase, and ATP synthase subunits. However, the primary transcripts of these genes need to be processed by inserting or removing uridylate residues to create functional mRNAs [39, 40]. Maxicircle transcripts have to be edited to create functional open reading frames. This editing process depends on the templates encoded by minicircles known as guide RNAs, which are responsible for nearly 60% of mRNA synthesised* de novo* [41]. The great variety of guide RNAs necessary to extensively edit maxicircle transcripts is a reasonable explanation for the large number of minicircle copies in comparison with the maxicircle repertoire in the kDNA network [42].

Despite the high heterogeneity of the minicircle population, a conserved region has been identified, to which the origins of replication are localised. Replication specifically occurs in the nuclear S phase, involving the participation of crucial proteins that support the process, such as polymerases, ligases, and topoisomerases [43]. In the early steps, minicircles are released from the network by topoisomerase II and replicate as free molecules. The minicircles are then closed covalently, forming a network again, but a continuous gap or nick can be observed until the replication of all molecules [41]. Another characteristic of minicircles is bent DNA structures consisting of multiple adenines sequences (5–26 bp) that participate in network organisation [39, 44].

### 3.3. Role in Bioenergetics

Trypanosomatid bioenergetics present remarkable differences from mammalian cells, such as the compartmentalisation of several steps of glycolysis into an organelle named the glycosome and mitochondrial ETC differences, accounting for the great majority of reports on* T. brucei *[45]. Due to their complex life cycles, trypanosomatids adapt to the environment in different hosts, reflecting the functional plasticity of the mitochondrion observed between the parasitic forms [3, 41, 46, 47].

As in higher eukaryotes, mitochondrial respiration occurs via the electron transport chain, which is composed of four integral enzyme complexes in the mitochondrial inner membrane: NADH-ubiquinone oxidoreductase (complex I), succinate-ubiquinone oxidoreductase (complex II), ubiquinolcytochrome c oxidoreductase (complex III or cytochrome bc1), and cytochrome oxidase (complex IV or cytochrome a3), with ubiquinone (coenzyme Q) and cytochrome c functioning as electron carriers between these complexes. Complexes I, III, and IV function as H+ pumps that generate a proton electrochemical gradient that drives ATP synthesis via the reversible mitochondrial ATP synthase (complex V), which couples the processes of respiration and phosphorylation [29, 48, 49].

*T. brucei *bloodstream forms are essentially glycolytic, living in an environment that presents high glucose levels. In this life stage, many tricarboxylic acid (TCA) cycle enzymes and cytochromes are not expressed in the mitochondrion, affecting energy production [45, 50]. However, F_0_-F_1_ ATP synthase and consequently the mitochondrial membrane potential (MMP) are still preserved, suggesting basal uncoupled activity in the organelle [51]. The mitochondrion of insect forms is much more functional, perhaps due to the large amounts of ETC substrates in the tsetse fly midgut [46]. This hypothesis also fits* T. cruzi* very well. Our group showed that epimastigotes' ETC is much more efficient than that of bloodstream trypomastigotes, confirming the functional adaptation of the parasite to the host substrates' availability [47].

Among the ETC substrates, succinate plays an essential role in trypanosomatids [52, 53]. In one of the most remarkable mitochondrial studies in these protozoa, Vercesi and colleagues [54] detailed the kinetics of succinate-sensitive oxygen uptake in digitonin-permeabilised* T. cruzi* epimastigotes and also described ETC stages 3 and 4. The oxidation of succinate by complex II leads to the transfer of electrons to complex III via ubiquinone, as occurred in higher eukaryotes. The activity of complexes II–IV was demonstrated in late 1970s in these protozoa, but the presence of functional complex I is still controversial [55, 56].

Curiously, rotenone-independent oxygen uptake has been described in these protozoa, with phenotypic effects observed only at high concentrations of this inhibitor [57]. Although the occurrence of NADH oxidation in* T. brucei *mitochondria is well known, no experimental data have confirmed its participation in respiration processes, even after the prediction of 19 complex I subunits in these parasites, including subunits that are involved in redox reactions [56]. In* T. cruzi *and* L. donovani*, oxygraphic studies revealed that pharmacological inhibition or the presence of natural subunit deletions does not affect oxygen consumption [52, 58]. All of these data indicate important differences in complex I subunits between trypanosomatids and other eukaryotes [56].

Interestingly, KCN, a complex IV inhibitor, does not completely abolish the respiratory rates of* T. brucei*,* T. cruzi,* and* L. donovani*, indicating the existence of a terminal oxidase that is an alternative to cytochrome oxidase. In* T. brucei*, this alternative oxidase (AOX) has been well characterised, with its three-dimensional structure being solved by X-ray crystallography [59]. AOX is a diiron protein that catalyses the four-electron reduction of oxygen to water by ubiquinol. AOX plays a critical role in the bloodstream forms of African trypanosomes, and its expression and amino acid sequence are identical in HAT-causing and non-human infective trypanosomes [60]. In trypanosomatids, the activity of salicylhydroxamic acid, an AOX inhibitor, was observed in both* T. brucei* and* T. cruzi*, suggesting a role in the organisms' energetic metabolism [3, 60]. In contrast, no effect of this inhibitor was detected in cyanide-insensitive* L. donovani* respiration, reinforcing the idea that the exact participation of AOX remains unclear and must be further investigated in trypanosomatids [61].

### 3.4. Role in Oxidative Stress

The single mitochondrion is one of the major sources of ROS in trypanosomatids, even under physiological conditions. Interestingly, these reactive species could play different roles in the parasites, involving signalling or cytotoxicity, and the cellular strategy for scavenging these species is crucial for protozoan survival [62–64]. Inside the parasites' organelle, the main site of ROS generation is the ETC complexes, except for* T. brucei* bloodstream forms. During mitochondrial respiration, part of the oxygen is reduced to superoxide anions and subsequently to hydrogen peroxide and hydroxyl radicals [65]. These species can cross the mitochondrial membranes and spread through the cytosol and other organelles, culminating in interference in biosynthetic pathways and deleterious consequences [62].

Complex I presents low NADH dehydrogenase activity, justifying the limited generation of superoxide observed in* T. brucei* procyclics and* T. cruzi* epimastigotes [58, 66]. The production of superoxide by rotenone-treated* L. donovani* promastigotes reinforces the necessity of further studies on the biological function of complex I in these parasites. Additionally, the involvement of complex II in ROS generation has been described in parasites treated with the specific inhibitor thenoyltrifluoroacetone [67]. However, there is no doubt that the most prominent ROS source in trypanosomatids is complex III [62, 66, 67]. Additionally, complex IV is not an electron leakage point in the ETC in trypanosomatids or even in higher eukaryotes. Treatment with salicylhydroxamic acid (SHAM) impairs complex IV, compromising electron flow and favouring electron escape from oxygen [62]. Our group reported that* T. cruzi* trypomastigotes present high activity for complexes II-III and low activity for complex IV, which correlates with the high ROS amounts detected in bloodstream forms in comparison with epimastigotes [47]. It was proposed that the AOX described in* T. brucei*, coexisting with complex IV, could play a role in ROS scavenging by the removal of excess reducing equivalents. The inhibition of this oxidase by SHAM confirmed this hypothesis, leading to an increase in ROS production within the protozoan mitochondrion [68].

To control the ROS concentration, pathogenic trypanosomatids present mitochondrial antioxidant defences. However, several differences can be observed in relation to mammals. Among the peculiarities of the protozoan antioxidant repertoire, the presence of an iron-superoxide dismutase and a selenium-independent glutathione peroxidase stands out, as these features are described in* T. brucei*,* T. cruzi,* and several species of* Leishmania* [61]. Surprisingly, the role of iron-superoxide dismutases is distinct among trypanosomatids. In* T. brucei*, these enzymes are not essential for the survival of bloodstream trypomastigotes, most likely due to the low ROS amounts produced by the rudimentary mitochondrion of this parasitic form [69]. In contrast,* T. cruzi *metacyclic trypomastigotes and* L. donovani* amastigotes express iron-superoxide dismutase isoforms in high amounts, indicating a possible relationship between the protozoan antioxidant system and host susceptibility to the infection [70, 71]. Moreover, thiol-based redox metabolism in these parasites involves a dithiol named trypanothione, formed by the conjugation of two glutathione molecules and one spermidine, and its corresponding reductase, a mitochondrial isoform already described in* T. cruzi* [64]. Peroxiredoxins, and especially tryparedoxin peroxidase, are also crucial to hydrogen peroxide detoxification, together with trypanothione reductase and tryparedoxin [72]. Interestingly, an increase in the expression of cytosolic and mitochondrial isoforms of tryparedoxin peroxidase in benznidazole-resistant* T. cruzi* was previously reported, reinforcing the importance of the antioxidant system for the infection outcome [73].

**Figure 2:**
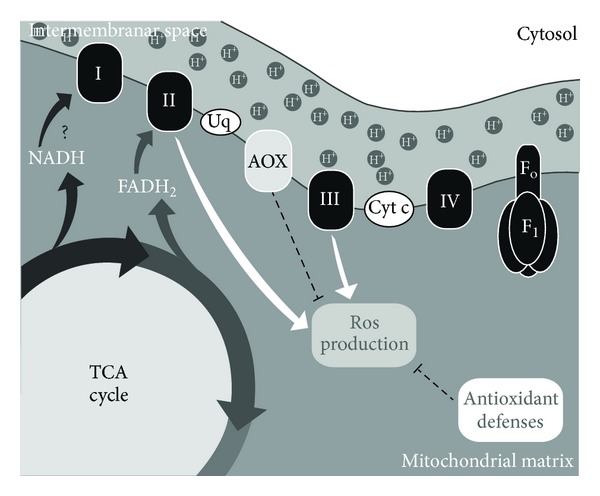
Mechanistic overview of oxidative stress in the trypanosomatid mitochondrion. Complexes II and III are the main sources of ROS, and complex I is not functional in these protozoa. AOX and antioxidant enzymes scavenge these reactive species within the organelle. White arrows: ROS generation and dashed lines: ROS scavenger.

Depending on their concentration, ROS can act as signalling molecules. The detoxification of these species by pathogenic trypanosomatids represents a crucial step in the success of the host-parasite interaction because ROS production is one of the mammalian mechanisms used to control the infection [74]. Recently, Piacenza and coworkers [75] demonstrated mitochondrial redox homeostasis in* T. cruzi* and found that its modulation by antioxidant defences (cytosolic and mitochondrial peroxiredoxins and trypanothione synthetase) contributes to the parasite's virulence, facilitating progression of the infection to the chronic phase. Additionally, Nogueira and colleagues (2011) reported that heme-induced ROS formation favours epimastigote proliferation through the activation of calmodulin kinase II and that this phenotype is regulated by treatment with exogenous antioxidants [76]. This finding indicates that an oxidative stress stimulus is necessary for cell cycle maintenance, at least in* T. cruzi*, and is fundamental to better comprehension of the regulation processes involved.  Figure 2 summarises mitochondrial ROS production in trypanosomatids.

### 3.5. Role in Cell Death

The existence of programmed cell death (PCD) in unicellular organisms has been a much-debated subject in the last two decades, as the precise molecular mechanisms that trigger death in protozoan parasites are still poorly comprehended. Despite the absence of strong evidence, an altruistic hypothesis has been proposed for trypanosomatids and other protists [32]. In fact, certain typical apoptotic hallmarks have been found, especially in pathogenic trypanosomatids. However, due to the lack of certain crucial molecular events, the existence of PCD in protozoa is still unconfirmed, so the term “apoptosis-like” is more suitable [32, 77].

Among PCD features, the proteolytic activity of caspases should be highlighted. These proteases have very specific substrates, and their cleavage represents a key step in the execution of apoptosis [78]. However, the orthologues of caspases that are present in pathogenic trypanosomatids, named metacaspases, demonstrate no involvement in cell death [79, 80]. In* Leishmania*, metacaspases are mitochondrial, but proteolysis has not been observed in parasites under oxidative stress [79]. In* T. cruzi*, the overexpression of metacaspase-3 and metacaspase-5 indicates their participation in cell cycle regulation and metacyclogenesis [81]. More investigation is necessary to clarify the exact role of metacaspases in unicellular organisms.

Most of the reports examining cell death in protozoa have evaluated the involved pathways under nonphysiological conditions (physical or chemical stresses). The mitochondrion plays a central role in this process, and alterations such as mitochondrial swelling and membrane depolarisation are the most recurrent signs of cell death [29, 77, 82–84].

As already discussed, the high ROS amounts produced by ETC impairment lead to severe deleterious effects in trypanosomatids. In this scenario,* T. cruzi* incubation in the presence of human sera induces important mitochondrial dysfunction and parasite death, a phenotype reverted by iron superoxide dismutase [85]. Similar results were observed in* T. brucei* and* L. donovani* after treatment with ROS inducers. Apoptotic-like features, including a loss of the MMP, were detected, and this phenotype was prevented by pretreatment with the ROS scavengers glutathione and* N*-acetylcysteine [67, 86]. Moreover,* T. brucei* AOX overexpression reduces ROS generation and consequently prevents the appearance of cell death phenotypes [68, 87].

### 3.6. Proteomic Analysis

Due to the posttranscriptional gene regulation of trypanosomatids, high-throughput proteomics have become essential for protein expression analysis and the validation of genomic annotations [88]. Nontranslated mRNA detection in* T. cruzi* also confirmed the limitation of RNA-based techniques in evaluating the protozoan's gene expression [89]. The proteomic map of pathogenic trypanosomatids has been assessed for the identification of virulence factors and stage-specific proteins and even for the characterisation of immunogenic molecule candidates for vaccines or diagnosis. In the last decade, subcellular proteomic studies have investigated enriched fractions of different organelles from these parasites, including the mitochondrion [88, 90]. This approach increases the number of proteins identifications in the desired fraction, increasing the coverage of the desired organellar content [91].

Different proteomic strategies have been employed in the investigation of the mitochondrial protein profile in trypanosomatids [90]. Mass spectrometry analysis of the mRNA editing mechanism presented in the mitochondria of* T. brucei* described 16 proteins involved in this process. The evaluation of mitochondrion-enriched fractions of* T. brucei* also led to the identification of several mitochondrial proteins, and especially ETC multiprotein complexes, including a unique oxidoreductase complex present only in kinetoplastids [92, 93]. Subsequently, many other proteins related to the TCA cycle, *β*-oxidation, and amino acid proteolysis were identified in procyclic but not in bloodstream trypomastigotes, reinforcing* T. brucei* mitochondrial plasticity [94]. In 2009, a shotgun approach was used to assess both the soluble and the hydrophobic proteomic content of the* T. brucei* mitochondrion [95]. This study led to the identification of 1,000 mitochondrial proteins, nearly 25% of which needed to have their function and localisation confirmed to exclude purification artefacts. More recently, label-free quantitative mass spectrometry was employed for the characterisation of* T. brucei* mitochondrial outer membrane [40]. Interestingly, 82 proteins were identified, of which approximately 36% are specific to trypanosomatids, but, to date, these proteins have unknown function. Knockdown assays of three of the proteins demonstrated their participation in the regulation of mitochondrial shape [40]. Additionally, proteomic characterisation of mitochondrial ribosomes was performed for procyclic forms of* T. brucei*, and more than 130 proteins were identified to be associated with the ribosomal structure by liquid chromatography and tandem mass spectrometry (LC-MS/MS) [96].

In* T. cruzi*, the specific mitochondrial protein profile has not yet been investigated. Atwood and colleagues (2005) [71] performed one of the most complete studies on this parasite's proteomics, describing the protein content of different developmental stages. Using a shotgun approach, 2,784 proteins were identified, with 838 detected in all parasitic forms, and a hypothetical annotation was presented for a substantial proportion. Among these identifications, several mitochondrial molecules, such as antioxidant enzymes and chaperones, were described, and their expression in the different parasitic forms evidenced adaptations to host environments. A large subcellular study by Ferella and coworkers [91] reported the expression of nearly all enzymes from the TCA cycle and succinate dehydrogenase subunits in the mitochondrion-enriched fraction. It is important to mention that the described mitochondrial proteins were not identified in a large-scale study by the Atwood III group [71], reinforcing the necessity of subfractionation to increase the number of identifications in specific organelles. In parallel, differential proteomic analyses of parasites treated with drugs were performed and indicated remarkable alterations in the mitochondrial protein content, confirming previous ultrastructural evidence [82, 97, 98]. Mass spectrometry was employed to investigate the drug resistance-related pathways in the parasite, revealing many mitochondrial proteins, such as chaperones, proteases, and antioxidant enzymes, are highly expressed in the resistant phenotype [99]. Recently, our group suggested that the mitochondrial isoform of the gluconeogenesis-related enzyme phosphoenolpyruvate carboxykinase (gi | 1709734) is a promising drug target based on proteomic analysis. The sequence differences between the parasitic and the human enzymes and their substrate specificity indicate that this molecule is a good candidate for drug intervention [100].

The profile of mitochondrial proteins in parasites of the genus* Leishmania* was first assessed in 2006. A two-dimensional electrophoretic analysis of mitochondrion-enriched fractions from* L. infantum* revealed several well-known mitochondrial proteins, and especially chaperones, whose localisation was confirmed by GFP-protein detection by fluorescence microscopy [90, 101]. In* L. donovani*, isobaric tagging for relative and absolute protein quantitation followed by an LC-MS/MS approach supported the hypothesis that changes in energetic metabolism are directly involved in parasite differentiation, as mitochondrial proteins related to the TCA cycle and oxidative phosphorylation are modulated during the parasite's life cycle [102]. The supplementary data in Supplementary Material available online at http://dx.doi.org/10.1155/2014/614014 summarize the proteomic findings in the mitochondrial profile of pathogenic trypanosomatids.

### 3.7. The Organelle as a Drug Target

The identification of a drug target in a pathogen requires that the target be either absent or at least substantially different in the host. Using metabolic systems that are very different from those of the host, parasites can adapt to the low oxygen tension present within the host animal. Most parasites do not use the oxygen available within the host to generate ATP but rather employ anaerobic metabolic pathways. Phylogenetically, trypanosomatids branch out relatively early relative to the higher eukaryotes. These organisms' cellular organisation is significantly different from that of the mammalian cells, and, thus, the existence of biochemical pathways unique to these pathogens is expected [103].

The fact that kinetoplastids have a single mitochondrion, rudimentary antioxidant defences, and a set of alternative oxidases indicates that this organelle is a potential candidate for drug intervention. In addition, several metabolic pathways are common to all pathogenic trypanosomatids, so, in principle, finding a single drug that is useful against all trypanosomatid diseases is a reasonable expectation. However, to date, this has not been the case, most likely due to the diversity of surroundings inside the parasite's hosts. African trypanosomes live in the bloodstream and cerebrospinal fluid,* T. cruzi* lives in the cytosol of various cell types, and* Leishmania* spp. lives within the phagolysosomes of macrophages.

**Figure 3:**
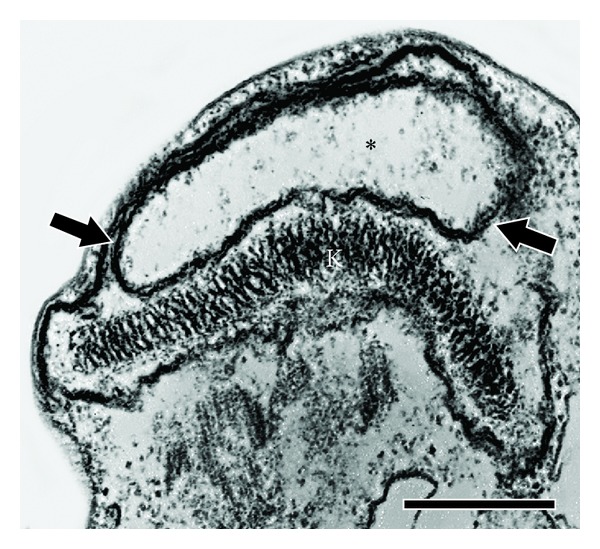
Common drug effects on the pathogenic trypanosomatid mitochondrion. Treatment with a naphthoquinone leads to mitochondrial swelling (asterisk) and the appearance of concentric membranar structures inside the organelle (arrows). K: typical morphology of the kDNA network. Bar: 200 nm.

The mitochondrion represents the most recurrent target, and the intensity of the alterations in this organelle is time dependent and varies with the compound employed [30, 104, 105]. Numerous articles point to the mitochondrion as a drug target in trypanosomatids, primarily based on transmission electron microscopy analysis and MMP evaluation using flow cytometry [29, 83, 106, 107]. As an example, the ultrastructural effect of a naphthoquinone on* T. cruzi* mitochondria can be observed in Figure 3. It is important to keep in mind, however, that induced mitochondrial alterations may be due to either a primary effect directly acting on this organelle or secondary lesions caused by a loss of cellular viability triggered by another cell component or metabolic pathway. Several other classes of compounds also interfere with the ultrastructure and physiology of the mitochondria of trypanosomatids such as sterol biosynthesis inhibitors (SBIs). Trypanosomatids have a strict requirement for specific endogenous ergosterol and analogs and cannot use the supply of cholesterol present in the mammalian host. One of the characteristic ultrastructural effects of SBIs on trypanosomatids is a marked swelling of their single giant mitochondrion, correlated with the depletion of the endogenous parasite sterols, which can lead to cell lysis [108–112]. Epimastigotes of* T. cruzi* treated with ketoconazole plus the lysophospholipid analogue edelfosine presented also severe mitochondrial swelling, with a decrease in electron density of its matrix and appearance of concentric membranar structures inside the organelle [113]. The group of Urbina has shown that* T. cruzi* mitochondrial membranes, in contrast to those of vertebrate cells, are indeed rich in specific parasite's sterols, which are probably required for their energy transducing activities [114, 115].

The mitochondrial metabolism of* Leishmania* spp. amastigotes and promastigotes,* T. cruzi* trypomastigotes and epimastigotes, and* T. brucei* procyclic forms is similar [53]. The inhibition of certain potential targets is associated with triggering apoptosis-like effects by MMP impairment and/or ROS production. The mitochondrial targeting of drugs may rely on free-radical production and/or calcium homeostasis [116].

Different potential targets can be identified in trypanosomatid mitochondria due to their unique characteristics in comparison with their mammalian counterpart: kDNA, topoisomerases, ETC and related enzymes, and RNA editing [30, 105].

Several drugs induce kDNA disorganisation such as diaminobenzidine, geranylgeraniol and vinblastine induce mitochondrial swelling and irregularly shaped kDNA [107, 117]. In trypanosomatids, growing evidence supports kDNA as the primary target of aromatic diamidines [118]. Ultrastructural and flow cytometric studies have shown that aromatic diamidines and reversed amidines target the* T. cruzi* mitochondrion-kinetoplast complex by interference with the MMP [119, 120]. In* T. brucei*, bloodstream forms exhibit a partial or even complete loss of kDNA, termed dyskinetoplastidy (Dk) and akinetoplastidy (Ak), respectively, which can be induced in the laboratory by DNA-binding drugs such as acriflavine or ethidium bromide [121]. In nature, most* T. brucei* strains contain a kinetoplast, and RNAi assays show that knockdown of kDNA replication and editing proteins is lethal to bloodstream forms [121], suggesting that the kinetoplast is a valid drug target. Moreover, Jensen and Englund [122] reported that minicircle replication is the most vulnerable target of ethidium bromide, which is still used to treat trypanosomiasis in African cattle [123]. Because the kinetoplast has no counterpart in other eukaryotes, complex kDNA replication and segregation present a potential drug target.

DNA topoisomerases are a well-studied mitochondrial target. These enzymes are involved in essential processes, such as DNA replication, transcription, recombination, and repair, and have been used as chemotherapeutic targets in bacterial diseases. DNA topoisomerases are broadly classified as type I, which cleaved single-stranded DNA, and type II, which acted on double-stranded DNA [124]. Two classes of drugs target topoisomerases: poisons (class I) which stabilise the DNA-enzyme complex, resulting in DNA breakdown, and catalytic inhibitors (class II) which compete with ATP for binding to the catalytic site interfering with the enzyme's function [117, 125]. Topoisomerase I purified from* T. cruzi* and* L. donovani* was found to be independent of ATP [126, 127]. In* T. brucei*, this enzyme is composed of two subunits encoded by two genes: one for the DNA-binding domain and a second for the C-terminal catalytic domain [128]. Topoisomerase II genes have been described in* T. brucei*,* T. cruzi*,* L. donovani,* and* L. infantum* [129, 130]. Interestingly, topoisomerase II from* T. brucei* and* L. donovani* exhibits both ATP-dependent and ATP-independent activities. The treatment of* T. brucei*,* T. cruzi,* and* L. donovani* with camptothecin (an inhibitor of eukaryotic DNA topoisomerase I) induces both nuclear and mitochondrial DNA cleavage and covalent linkage to the protein, which is consistent with the existence of drug-sensitive topoisomerase I activity in both compartments [131]. In contrast to other eukaryotic topoisomerases,* L. donovani* topoisomerase is distinct from that of other eukaryotes with respect to its biological properties and sensitivity to drugs [127, 132].* L. donovani* promastigotes and amastigotes present different sensibility to topoisomerase I inhibitors [133–136]. In* T. cruzi*, topoisomerase II is highly expressed in the replicative forms of the parasite, accounting for the trypanocidal effect of the specific inhibitors clorobiocin, novobiocin, ofloxacin, and nalidixic acid [137–139]. Ultrastructural alterations were also observed in* L. amazonensis* promastigotes treated with these inhibitors [138].

The ETC in trypanosomatids has peculiarities that make its components a promising target, given that MMP maintenance is vital for cell survival. Studies have shown that the loss of MMP induced by drugs is associated with pathogenic trypanosomatid death [67, 83, 140, 141]. Most of the studies on ETC as a drug target in trypanosomatids have been performed with* L. donovani* promastigotes. Pentamidine also induced a rapid collapse of the mitochondrial inner membrane potential of* L. donovani* promastigotes [142]. The association of resistance to pentamidine with mitochondrial alterations was based on studies with its fluorescent analogue DB99 in which drug accumulation in the kinetoplast was observed with wild-type* L. donovani* but not with a resistant strain [143]. Mehta and Saha [67] observed that concurrent inhibition of respiratory chain complex II with pentamidine administration increases the cytotoxicity of the drug. Inhibitors of respiratory chain complexes I (rotenone), II (-noyltrifluoroacetone (TTFA)), and III (antimycin A) resulted in MMP dissipation, ROS production, and the induction of apoptosis-like effects. Additionally, 4,4′-bis((tri-n-pentylphosphonium)methyl)benzophenone dibromide and sitamaquine also target complex II, causing dramatic mitochondrial compromise, including organelle swelling, a decrease in cytoplasmic ATP, ROS production, inhibition of the oxygen consumption rate, and impairment of the cell cycle in* L. donovani* [144, 145]. Meanwhile, tafenoquine (a primaquine analogue) and miltefosine (a lysophospholipid analogue) inhibit complexes III and IV, respectively, leading to a similar phenotype [146, 147].

Because AOX does not exist in hosts, this enzyme has been proposed as an innovative target for antitrypanosomatid drug development, and related attempts have been reported in the literature [148]. Ascofuranone, an antibiotic isolated from the fungus* Ascochyta viciae*, has been reported as effective against African trypanosomes* in vitro*, and ubiquinol oxidase was identified as the drug's molecular target [148, 149]. It was reported that treatment with ascofuranone led to the appearance of PCD-like features in* T. b. rhodesiense* bloodstream forms [87].

Mitochondrial RNA editing is a vital and unique process that occurs in the mitochondria of trypanosomatids. This specificity makes RNA editing a potential target for new antiparasitic drugs. In* T. brucei*, an RNA editing process has been described. The mRNAs encoding the cytochrome system are mainly edited in the procyclic forms, whereas the mRNAs encoding the NADH dehydrogenase complex are edited in the bloodstream forms [150]. This differential RNA editing observed in the parasite has been less studied in other trypanosomatids. Kim et al. [151] examined the differential expression of subunit II of cytochrome oxidase, but, in contrast to* T. brucei*, no differences were observed in the mRNA levels of this enzyme in either* T. cruzi* insects or mammalian stages. Furthermore, the contribution of the RNA editing process to mitochondrial functional plasticity cannot be excluded. Presently, this possibility should be considered as a hypothesis, and additional studies are needed for confirmation [152]. In this context, Liang and Connell [153] employed high-throughput screening to identify specific inhibitors of RNA editing. Five compounds were identified (GW5074, mitoxantrone, NF 023, protoporphyrin IX, and D-sphingosine), which proved to be inhibitors of insertional editing. More specifically, GW5074 and protoporphyrin IX inhibited the editing process at the level of endonuclease cleavage, which begins the editing process [153]. Recently, another potential target in the RNA editing process was proposed, and inhibition of the RNA ligase KREL1 was described in* T. brucei* [154].

## 4. Conclusions

In the last decade, the mechanisms of action of numerous drugs have been found to be involved directly or indirectly in mitochondrial metabolism, leading this organelle to become a promising target in the treatment of different diseases. In pathogenic trypanosomatids, the presence of a single mitochondrion, together with its peculiarities, such as the existence of AOX and unique antioxidant defences, attributes a crucial role to the organelle in the development of novel active compounds. Moreover, the morphological and functional plasticity of the mitochondrion during these parasites' life cycles also represent a fundamental step in protozoan adaptations to the host environment. Variation in the efficiency of the respiratory machinery could compromise the redox balance and culminate in ROS generation. Despite their well-known cytotoxic effect, the role of ROS in these protozoa is complex. Depending on the concentration, these reactive species lead to the parasites' death or participate in their cell signalling and proliferation. Thus, better comprehension of oxidative regulation could support new perspectives on trypanosomatid-targeting chemotherapy.

## Supplementary Material

In the supplementary table 1, it is presented the list of mitochondrial proteins identified by proteomic approaches in *T. brucei*, *T. cruzi* and *Leishmania* spp. This list is separated by specie, and each list showed the comparison of the identifications of two references. *T. brucei*: Acestor et al 2009 and Panigrahi et al 2009; *T. cruzi*: Atwood et al 2005 and Nakayasu et al 2012; and *Leishmania* spp.: Paape et al 2010 and Nirujogi et al. 2013.
