# Supplementary material for: The Double-Edged Sword in Pathogenic Trypanosomatids: The Pivotal Role of Mitochondria in Oxidative Stress and Bioenergetics
Source: Biomed Res Int. 2014 Mar 31;2014:614014. doi: 10.1155/2014/614014 (PMC3988864; doi:10.1155/2014/614014)
Supplement: Supplementary file 1 — In the supplementary table 1, it is presented the list of mitochondrial proteins identified by proteomic approaches in T. brucei, T. cruzi and Leishmania spp. This list is separated by specie, and each list showed the comparison of the identifications of two references. T. brucei: Acestor et al 2009 and Panigrahi et al 2009; T. cruzi: Atwood et al 2005 and Nakayasu et al 2012; and Leishmania spp.: Paape et al 2010 and Nirujogi et al. 2013. [file 614014.f1.pdf]

Gene ID	Protein description
LmjF25.2010	2,4-dihydroxyhept-2-ene-1,7-dioic acid aldolase, putative
LinJ.35.0050<br>LdBPK_350050.1<br>LmjF35.0050	2-oxoisovalerate dehydrogenase beta subunit, mitochondrial precursor, putative
LdBPK_312320.1<br>LmjF31.2250	3,2-trans-enoyl-CoA isomerase, mitochondrial precursor, putative
LdBPK_290010.1	3,2-trans-enoyl-CoA isomerase, mitochondrial precursor-like protein
LmjF30.3190<br>LinJ.30.3230	3-hydroxy-3-methylglutaryl-CoA reductase (HMGR)
LmjF23.0690	3-ketoacyl-CoA thiolase, putative
LinJ.25.1850	3-oxo-5-alpha-steroid 4-dehydrogenase, putative
LmjF31.2970	acetyl-CoA carboxylase, putative
LinJ.04.1250	actin (ACT)
LmjF27.0290	acyl carrier protein, putative (ACP)
LmjF07.0460<br>LdBPK_070150.1	acyl-CoA dehydrogenase, mitochondrial precursor, putative
LinJ.19.0200<br>LdBPK_190200.1<br>LmjF19.0210	ADP,ATP carrier protein 1, mitochondrial precursor, putative,ADP/ATP translocase 1, putative (ANC2)
LmjF14.0990<br>LdBPK_141050.1	ADP/ATP mitochondrial carrier-like protein

LinJ.25.1160<br>LdBPK_251160.1<br>LmjF25.1120	aldehyde dehydrogenase, mitochondrial precursor (ALDH2)
LdBPK_364000.1	aminomethyltransferase, mitochondrial precursor, putative
LmjF34.0070<br>LinJ.34.0070	ascorbate peroxidase (APX)
LinJ.36.2130<br>LdBPK_362130.1<br>LdBPK_362140.1<br>LinJ.32.1940	chaperonin HSP60, mitochondrial precursor
LmjF18.0680<br>LdBPK_180700.1	citrate synthase, putative
LinJ.30.0760<br>LmjF30.0730	co-chaperone GrpE, putative
LmjF09.1490<br>LdBPK_110490.1	cytochrome b5-like protein, putative
LmjF04.1130<br>LmjF20.0840<br>LdBPK_030090.1	cytochrome c oxidase assembly factor, putative
LinJ.03.0720<br>LdBPK_030720.1	cytochrome c oxidase copper chaperone, putative
LinJ.23.0420	cytochrome c oxidase subunit 10, putative
LdBPK_367350.1	cytochrome c oxidase subunit I
LmjF12.0670<br>LinJ.12.0620	cytochrome c oxidase subunit IV
LmjF26.1710<br>LinJ.26.1690	cytochrome c oxidase subunit V, putative
LdBPK_212080.1	cytochrome c oxidase subunit VI, putative

LinJ.25.1170	cytochrome c oxidase VII, putative
LmjF31.1570<br>LinJ.31.1600	cytochrome c oxidase VIII (COX VIII), putative
LmjF16.1310<br>LdBPK_161390.1	cytochrome c, putative
LinJ.07.0210<br>LdBPK_070210.1<br>LmjF07.0060	cytochrome c1, heme protein, mitochondrial precursor, putative
LdBPK_150050.1	cytochrome-b5 reductase, putative
LmjF03.0200	delta-1-pyrroline-5-carboxylate dehydrogenase, putative
LinJ.16.1340	diacylglycerol kinase, putative
LinJ.32.3510<br>LmjF32.3310	dihydrolipoamide dehydrogenase, putative (GCVL-2)
LinJ.15.1270	E2-like ubiquitin-conjugation enzyme (UFC1)
LmjF25.1770	enoyl-CoA reductase, putative (EnCR)
LinJ.28.0240<br>LdBPK_280240.1<br>LmjF28.0240	glycerol-3-phosphate dehydrogenase (FAD-dependent), mitochondrial
LmjF36.3800	glycine synthase, putative (GCVT1)
LinJ.36.4000	glycine synthase, putative (GCVT-2)
LmjF33.2730	guide RNA associated protein, GAP1, putative

LinJ.22.0520<br>LmjF22.0650	guide RNA associated protein, GAP2, putative
LmjF30.2480<br>LdBPK_302480.1	heat shock 70-related protein 1, mitochondrial precursor, putative
LinJ.15.0090<br>LmjF.15.0090<br>LmjF15.0090	heat shock protein HslVU, ATPase subunit HslU, putative
LmjF36.3990<br>LinJ.36.4180	hs1vu complex proteolytic subunit-like, threonine peptidase, Clan T(1), family T1B
LinJ.04.0260	hypothetical protein, conserved
LinJ.08.0340	hypothetical protein, conserved
LinJ.09.1070	hypothetical protein, conserved
LinJ.22.1330	hypothetical protein, conserved
LinJ.23.0870	hypothetical protein, conserved
LmjF.35.0100	hypothetical protein, conserved
LmjF04.0270	hypothetical protein, conserved
LmjF09.1010	hypothetical protein, conserved
LmjF36.0620	hypothetical protein, conserved
LmjF36.3890	hypothetical protein, conserved

LinJ.35.3670<br>LmjF35.3620	iron-sulfur cluster assembly protein, putative
LinJ.10.0310<br>LdBPK_100310.1<br>LmjF10.0290	isocitrate dehydrogenase [NADP], mitochondrial precursor, putative
LinJ.33.2680	isocitrate dehydrogenase, putative
LmjF18.0010	kinetoplast polyadenylation/uridylation factor 1
LmjF32.1140	kinetoplast polyadenylation/uridylation factor 2, putative
LinJ.36.2440<br>LmjF27.0630<br>LmjF33.2510<br>LmjF36.2310	kinteoplast poly(A) polymerase complex 1 subunit, putative,mitochondrial edited mRNA stability factor 1 subunit, putative
LinJ.01.0610	KREL1
LinJ.29.1000<br>LmjF29.0920	LETM1 and EF-hand domain-containing protein 1, putative
LinJ.35.4790<br>LmjF35.4720	lipoic acid containing carrier protein, putative (GCVH)
LdBPK_190350.1	lipoic acid synthetase, mitochondrial precursor, putative
LinJ.08.0330<br>LdBPK_080330.1<br>LmjF08.0320	mitochondrial associated ribonuclease, putative
LmjF36.2710<br>LdBPK_341130.1<br>LdBPK_362850.1	mitochondrial ATP-dependent zinc metallopeptidase, putative
LinJ.36.2850<br>LinJ.34.1130	mitochondrial ATP-dependent zinc metallopeptidase, putative,metallo-peptidase, Clan MA(E), Family M41
LinJ.02.0640<br>LinJ.19.1090<br>LdBPK_020640.1<br>LdBPK_191090.1	mitochondrial carrier protein, putative

LinJ.29.2890<br>LinJ.30.1110<br>LdBPK_292890.1<br>LdBPK_301110.1	mitochondrial carrier protein-like protein
LinJ.28.2220<br>LdBPK_282220.1<br>LmjF28.2080	mitochondrial DEAD box protein, putative
LinJ.08.0830<br>LdBPK_080830.1<br>LmjF08.0890	mitochondrial DNA polymerase beta
LmjF08.0900<br>LdBPK_080840.1	mitochondrial DNA polymerase beta-PAK, putative
LdBPK_130080.1	mitochondrial DNA polymerase I protein B, putative
LmjF14.0920<br>LdBPK_140980.1	mitochondrial DNA polymerase I protein C, putative
LdBPK_131370.1	mitochondrial DNA polymerase I protein D, putative
LmjF23.0680	mitochondrial DNA primase, putative (PRI1)
LdBPK_151310.1	mitochondrial DNA topoisomerase II
LdBPK_241180.1	mitochondrial DNA-directed RNA polymerase, putative
LinJ.36.0620<br>LdBPK_360620.1<br>LmjF36.0570	Mitochondrial elongation factor G, putative (MEFG)
LmjF04.0330<br>LdBPK_040320.1	mitochondrial exoribonuclease DSS-1, putative
LinJ.09.1190<br>LdBPK_091190.1	mitochondrial import inner membrane translocase subunit TIM17, putative,inner membrane preprotein translocase Tim17, putative
LmjF36.4450<br>LdBPK_364670.1	mitochondrial intermediate peptidase, putative

LmjF34.0160<br>LdBPK_340170.1	mitochondrial malate dehydrogenase (mMDH)
LmjF30.0780<br>LdBPK_300830.1	mitochondrial oligo_U binding protein TBRGG1, putative
LmjF35.4430<br>LdBPK_354490.1	mitochondrial phosphate transporter, putative
LmjF13.0870<br>LmjF21.0340<br>LmjF33.2610<br>LinJ.13.0760	mitochondrial processing peptidase alpha subunit, putative
LinJ.35.1390<br>LdBPK_351390.1<br>LdBPK_010670.1<br>LmjF35.1380	mitochondrial processing peptidase, beta subunit, putative,metallo-peptidase, Clan ME, Family M16
LinJ.14.1560	mitochondrial pyruvate carrier protein 2, putative
LmjF25.1540<br>LmjF25.1740<br>LmjF26.1140<br>LmjF31.0630	mitochondrial RNA binding complex 1 subunit, putative
LinJ.27.0980<br>LdBPK_270980.1	mitochondrial RNA binding protein 1,gBP21, MRP1
LmjF24.0830	mitochondrial RNA binding protein 1,mitochondrial edited mRNA stability factor 1,Mitochondrial poly(A) polymerase 1
LmjF09.1120	mitochondrial RNA binding protein 2
LinJ.09.1180<br>LdBPK_091180.1	mitochondrial RNA binding protein 2,MRP2, gBP25
LinJ.33.1320<br>LdBPK_230930.1<br>LmjF33.1250	mitochondrial RNA binding protein, putative
LdBPK_010610.1	mitochondrial RNA editing ligase 1
LinJ.21.2020<br>LdBPK_212020.1<br>LmjF21.1660	mitochondrial structure specific endonuclease I (SSE-1), putative

LdBPK_240730.1	mitochondrial translocase subunit, putative
LmjF05.0310	monothiol glutaredoxin, putative
LmjF36.0700	MP18 RNA editing complex protein, putative
LinJ.08.1080<br>LmjF08.1170	MRB1-associated protein, putative,guide RNA binding protein, putative
LinJ.32.3370<br>LmjF.32.3170	NADH dehydrogenase subunit NI8M, putative
LinJ.36.3680	NADH-ubiquinone oxidoreductase complex I subunit, putative
LinJ.05.0980<br>LdBPK_050980.1<br>LdBPK_170320.1<br>LdBPK_120730.1	NADH-ubiquinone oxidoreductase, mitochondrial, putative
LinJ.32.2590<br>LmjF32.2440	NUDIX hydrolase, putative,mitochondrial RNA binding complex 1 subunit, putative
LinJ.30.1030<br>LmjF30.0970	p22 protein precursor, putative
LmjF28.0980	P27 protein, putative (P27)
LmjF04.0820	peptide deformylase, putative (EMBL:AY353252) (metalloprotease-like protein)
LmjF34.4530	phosphatidylinositol 3-kinase (tor2), putative (TOR2)
LmjF19.1620	potassium voltage-gated channel, putative
LmjF26.1610<br>LdBPK_261590.1	proline oxidase, mitochondrial precursor-like protein

LmjF34.1250	PTP1-interacting protein, 39 kDa, putative
LmjF26.0030	pyridoxal phosphate containing glycine decarboxylase, putative (GCVP)
LmjF15.0280<br>LdBPK_150330.1<br>LinJ.15.0320	ribonucleoprotein p18, mitochondrial precursor, putative
LmjF34.3230	RNA editing associated helicase 2, putative (REH2)
LmjF28.2370	serine hydroxymethyltransferase (SHMT-L) (SHMT-L)
LmjF11.0100	seryl-tRNA synthetase, putative
LinJ.36.3100<br>LmjF36.2950	succinyl-CoA ligase [GDP-forming] beta-chain, putative
LinJ.33.2470<br>LdBPK_332470.1<br>LmjF33.2340	succinyl-coA:3-ketoacid-coenzyme A transferase, mitochondrial precursor, putative
LinJ.29.1940<br>LmjF29.1820	Tob55, putative
LinJ.24.1570<br>LmjF24.1500	translationally controlled tumor protein (TCTP), putative
LmjF26.1550<br>LdBPK_261530.1	trifunctional enzyme alpha subunit, mitochondrial precursor-like protein
LmjF29.1150	tryparedoxin (TXN2)
LinJ.31.2650	ubiquinol-cytochrome-c reductase-like protein
LmjF16.1065	ubiquitin fold modifier protein, putative (UFM1)

LinJ.02.0430<br>LmjF02.0460	voltage-dependent anion-selective channel, putative

Paape et al. 2010	Nirujogi et al. 2013
yes	no
yes	yes
yes	yes
no	yes
yes	yes
yes	no
no	yes
yes	no
no	yes
yes	no
yes	yes
yes	yes
yes	yes

yes	yes
no	yes
yes	yes
yes	yes
yes	yes
yes	yes
yes	yes
yes	yes
no	yes
no	yes
no	yes
yes	yes
yes	yes
no	yes

no	yes
yes	yes
yes	yes
yes	yes
no	yes
yes	no
no	yes
yes	yes
no	yes
yes	no
yes	yes
yes	no
no	yes
yes	no

yes	yes
yes	yes
yes	yes
yes	yes
no	yes
no	yes
no	yes
no	yes
no	yes
no	yes
yes	no
yes	no
yes	no
yes	no

yes	yes
yes	yes
no	yes
yes	no
yes	no
yes	yes
no	yes
yes	yes
yes	yes
no	yes
yes	yes
yes	yes
no	yes
yes	yes

no	yes
yes	yes
yes	yes
yes	yes
no	yes
yes	yes
no	yes
yes	no
no	yes
no	yes
yes	yes
yes	yes
no	yes
yes	yes

yes	yes
yes	yes
yes	yes
yes	yes
yes	yes
no	yes
yes	yes
no	yes
yes	no
yes	no
no	yes
yes	yes
no	yes
yes	yes

no	yes
yes	no
yes	no
yes	yes
no	yes
no	yes
no	yes
yes	yes
yes	yes
yes	no
yes	no
yes	no
yes	yes

yes	no
yes	no
yes	yes
yes	no
yes	no
yes	no
yes	yes
yes	yes
yes	yes
yes	yes
yes	yes
yes	no
no	yes
yes	no

yes	yes
